# Immunoprotective Effect of Seabuckthorn (*Hippophae rhamnoides*) and Glucomannan on T-2 Toxin-Induced Immunodepression in Poultry

**DOI:** 10.4061/2010/149373

**Published:** 2010-12-01

**Authors:** T. Ramasamy, C. Varshneya, V. C. Katoch

**Affiliations:** ^1^Department of Pharmacology and Toxicology, College of Veterinary and Animal Sciences, CSKHPKV, Himachal Pradesh Palampur 176062, India; ^2^Department of Veterinary Microbiology and Immunology, College of Veterinary and Animal Sciences, CSKHPKV, Himachal Pradesh Palampur 176062, India

## Abstract

The present investigation was undertaken to study the immunoprotective effect of seabuckthorn berries and glucomannan against T-2 toxin-induced immunodepression in 15-day-old chicks. T-2 toxin was produced in the laboratory by growing *Fusarium sporotrichioides* MTCC 2081 on wheat. T-2 toxin was fed to birds at 1 ppm level of the diet. The powdered seabuckthorn berries were added at 400 and 800 ppm levels, and glucomannan added at 1 g/kg of feed. All the treatments were continued up to 28 days. The immunoprotective effects of seabuckthorn and glucomannan were assessed by evaluating humoral immune reaction against NCD vaccine (haemagglutination test and immunoglobulin estimation), serum immunoglobulin levels, phagocytic index, and DTH reaction against DNFB between day 25 and day 28 of experiment. There was significant (*P* < .05) decrease in non-specific immunity in T-2 toxin-treated group as evidenced by a reduction in phagocytic index, DTH reaction, HI titer, and total serum Ig compared to the healthy control group. A significant increase (*P* < .05) in HI titer and total serum Ig was seen in seabuckthorn and glucomannan fed group. A significant (*P* < .05) increase in DTH reaction and non-specific immune response was seen in seabuckthorn and glucomannan fed birds. The present investigation revealed that the seabuckthorn alone protected the immunosuppressant action of T-2 toxin, but seabuckthorn and glucomannan in combination provided an additive protection against T-2 toxicity.

## 1. Introduction

Mycotoxins belong to various groups of structurally diverse secondary metabolites produced by toxigenic strains of several fungi. Mycotoxins elicit a wide spectrum of toxicological effects, such as immunosuppression [1]. T-2 toxin, a naturally occurring mycotoxin produced by several species of genus Fusarium, is a 3-hydroxy 4, 15 diacetoxy-8 (3-methyl butryloxy), 12, 13-epoxy trichothec-9-ene metabolite, that is, known to suppress the both cellular and humoral-mediated immune responses [2] and inhibit protein synthesis [3]. To date, investigation on the effects of T-2 toxin on immune system has been done mainly with laboratory animals and these studies showed that T-2 toxin-induced deleterious effects on immune system [4]. In poultry, T-2 toxin induces immunotoxicity, increased susceptibility to diseases, and subsequent loss in poultry production [5]. It is therefore important to provide immunoprotection against mycotoxin immune suppression in birds. In this study, plant-derived products such as seabuckthorn (a native shrub of dry temperate Himalayan region) and glucomannan (mycotoxin binder) were evaluated for immunoprotective action against T-2 toxin immunosuppression in broiler chickens.

## 2. Material and Methods

Two hundred and three (203), day-old broiler chicks (30–40 g) were procured from a local hatchery (Uttam Poultry Breeding Farm, Ghurkari, Kangra, H.P) and housed in battery brooders with *ad libitum* supply of feed and water. They were randomly distributed into seven groups (control, mycotoxin alone, mycotoxin + glucomannan, mycotoxin + seabuckthorn 400 ppm, mycotoxin + seabuckthorn 800 ppm, mycotoxin + seabuckthorn 400 ppm + glucomannan, mycotoxin + seabuckthorn 800 pppm + glucomannan) of 29 chicks each. The T-2 toxin was produced in the wheat using *Fusarium sporotrichioides* MTCC 2081 [6] and quantified by using thin layer chromatography [7].

The experiments were performed in accordance with the guidelines of Institutional Animal Ethical Committee of the College of Veterinary and Animal Sciences, CSK H.P. Agricultural University, Palampur, H.P. India. Broiler mash fed to chickens in this study was tested at the Animal Feed Analytical and Quality Control Laboratory, Veterinary College and Research Institute, Namakkal-637 001, Tamil Nadu (India) to ascertain that the feed was free of toxin binders, aflatoxins, and T-2 toxin. The ripen berries of seabuckthorn were collected from Agricultural Research Extension Centre, Kukumseri, Lahaul (H.P) India and shade dried and ground to obtain a fine powder. The purified glucomannan powder was procured from the Neospark, Drugs and Chemicals Private Limited, Hyderabad—500 082 A.P. India. 

The T-2 toxin culture material, glucomannan, and Seabuckthorn berries powder were incorporated into the broiler mash at 1 ppm, 1 g/kg feed, and 400 or 800 ppm, respectively, for 28 days. The birds were vaccinated against Newcastle disease (NDV) at 7 and 14 days of age by intraocularly administering one drop (10^6^ EID50/bird) of Newcastle disease virus Lasota strain vaccine (Venkey's Biological Limited, India).

## 3. Humoral Immune Responses

### 3.1. Antibody Titres Against NDV

 About 0.5 ml serum samples were collected from vaccinated birds on day 28 and heat-inactivated to destroy the complement by placing it in water bath at 56°C for 30 min. These serum samples were later subjected to haemagglutination inhibition (HI) test. Because the HI test requires the use of NCD (Lasota) antigen that has a known haemagglutination (HA), the HA unit of NCD (Lasota) antigen was first determined using the procedures of Chauhan and Roy [8]. Thereafter, HI test was performed as follows: a volume of 50 *μ*L of PBS (pH 7.2) was placed into each of the 12 wells in the first row of a 96-well U-bottom microtitre plate. Thereafter, 50 *μ*l test serum was added to the first well, mixed thoroughly, and transferred serially to the next ten wells by twofold serial dilution. From the 11th well, 50 *μ*l was taken and discarded to achieve equal volume in each well. Next, 50 *μ*l of 4-haemagglutination unit (4-HAU) of NCD antigen was added in eleven wells. The 12th well was kept as control. The plate was then incubated at room temperature for 15 min. Then, 50 *μ*l of 1% chicken RBCs suspension in PBS was added to each of the twelve wells. The plate was then incubated at room temperature for 30 min, after which results were recorded and expressed as reciprocal of log2 of titre.

### 3.2. Total Immunoglobulin (Ig) Estimation

Total serum immunoglobulin was estimated on day 28 after administration by using zinc sulphate turbidity test [9]. The serum total immunoglobulin values were expressed in g/dl.

### 3.3. Cell-Mediated Immune Responses

#### 3.3.1. Phagocytic Index

The metabolic activity of peritoneal phagocytes was assessed on day 27 using nitro blue tetrazolium (NBT) dye reduction test as described by Talwar [10] and Chauhan [11].

#### 3.3.2. Delayed-Type Hypersensitivity Test

 Cell-mediated immune response was assessed on day 25 posttreatment by delayed-type hypersensitivity reaction to 2, 4-dinitroflurobenzene (DNFB) by the method of Phanuphak et al. [12]. Seven birds from each group were randomly selected and featherless areas of about 10 cm^2^ were chosen on left and right lateral side of abdomen for DNFB application and cleaned with the help of alcohol. The DNFB (10 mg/ml) in acetone vehicle (0.25 ml) was applied on right side, and acetone (0.25 ml) was applied on left side, which served as control.

Two weeks later the sensitized birds were challenged by applying 0.25 ml of DNFB (1 mg/ml) on right side and 0.25 ml acetone on left side. The skin thickness was measured with the help of Vernier caliper at 0, 12, 24, 36, 48, 72 and 96 hrs post-challenge. The application area of skin was also examined for erythema, induration, ulceration and scab formation.

#### 3.3.3. Statistical Analysis

The data were analyzed using Graph Pad Instat version 3.00 for windows (Graph Pad Software, San Diego, California, USA, and http://www.Graphpad.com/) and the significant differences between mean values were determined using Tukey-Kramer Multiple comparisons test. The comparison was made at 5% levels of significance.

## 4. Results

### 4.1. Antibody Titres

A significant (*P* < .05) reduction in haemagglutination inhibition titers was observed in T-2 toxin-treated group in both vaccinated and unvaccinated birds when compared with the healthy controls. However, with the exception of the T + GM treatment, birds in the other unvaccinated treatment groups elicited significantly higher (*P* < .05) HI titers compared to toxin alone treated group. Corroborating this finding is the fact that among vaccinated birds, all treatment groups elicited significantly higher (*P* < .05) HI titers compared to birds in the toxin fed group. 

Another important finding is the fact that the T + SBT800 + GM treatment of both vaccinated and unvaccinated birds revealed significantly higher (*P* < .05) HI titers compared to healthy control birds (Table 1).

### 4.2. Total Immunoglobulin Estimation

On day 28 of experiment, both unvaccinated and vaccinated birds fed T-2 toxin had significantly lower (*P* < .05) total serum immunoglobulin (Ig) when compared to healthy controls, while both the vaccinated and unvaccinated birds in SBT 800 ppm plus GM plus toxin group revealed significantly higher (*P* < .05) total serum Ig compared to the healthy control birds (Table 2).

### 4.3. Phagocytic Index

**Figure 1:**
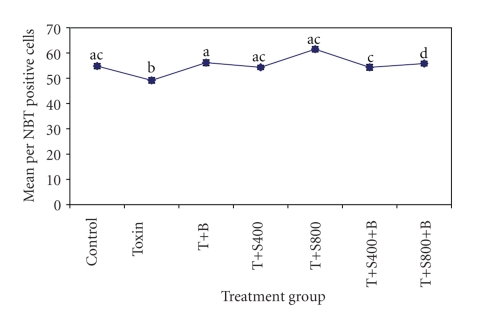
Effect of dietary supplementation of seabuckthorn berries (SBT) and glucomannan (GM, mycotoxin binder) on macrophage function test (per cent NBT positive cell) in T-2toxin-fed birds.

A significant (*P* < .05) decrease in the number of nitroblue tetrazolium positive cells were recorded only in the T-2 toxin-treated birds when compared to those in the healthy control group. However, a significant (*P* < .05) increase in the number of nitroblue tetrazolium positive cells were found in the rest of the treatment groups as compared to toxin-treated group. The NBT positive cells were found to be significantly (*P* < .05) increased in SBT 800 ppm plus GM plus toxin-fed group and SBT 400 ppm plus GM plus toxin-fed group when compared with the healthy control group (Figure 1).

### 4.4. Delayed-Type Hypersensitivity Reaction

#### 4.4.1. Mean Skin Thickness

**Figure 2:**
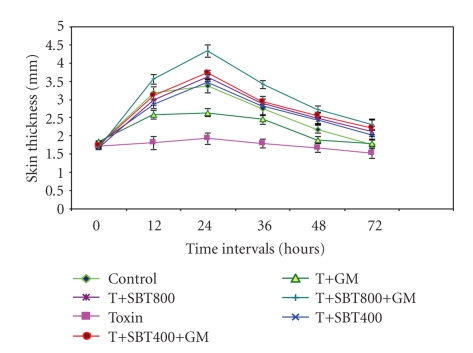
Effect of dietary supplementation of seabuckthorn berries (SBT) and glucomannan (GM, mycotoxin binder) on skin thickness (mm) in delayed-type hypersensitivity reaction in birds fed with T-2 toxin.

Cell-mediated immune response was evaluated employing delayed-type hypersensitivity reaction using DNFB and the skin thickness of abdomen region at 12, 24, 36, 48, and 72 hrs after challenge with DNFB in different groups was measured using Vernier calipers (Table 3). A significant (*P* < .05) increase in the skin thickness was recorded at 12, 24, and 36 hrs after-challenge in all treatment groups as compared to toxin-treated group. The SBT400 ppm plus GM plus toxin and SBT 800 ppm plus GM plus toxin-treated groups revealed significantly (*P* < .05) increased skin thickness at 24, 36, and 48 hrs after DNFB challenge when compared with healthy control birds. A significant (*P* < .05) decrease in the mean skin thickness of T-2 toxin-treated birds were observed at 12, 24, 36, 48, and 72 hrs as compared to healthy control birds (Figure 2).

#### 4.4.2. Gross Skin Lesion

**Figure 3:**
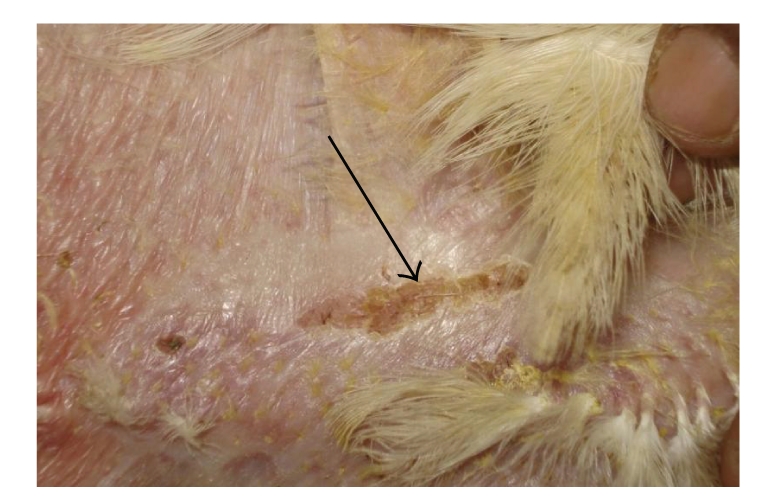
DTH reaction of skin (control): increased thickness and scab formation.

**Figure 4:**
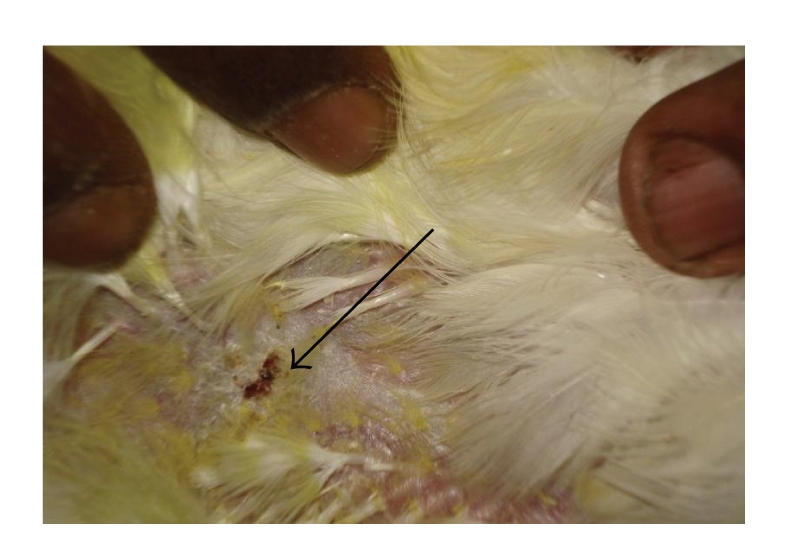
DTH reaction of skin (T-2 toxin fed bird): minimal skin thickness and scab formation.

**Figure 5:**
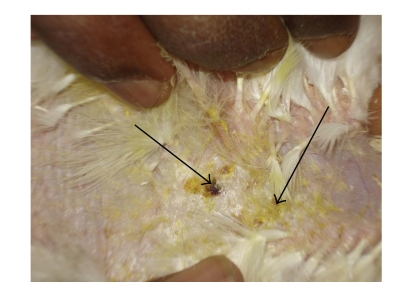
DTH reaction of skin (T-2 toxin plus GM fed bird): mild increase in skin thickness and scab formation compare to toxin-fed bird.

**Figure 6:**
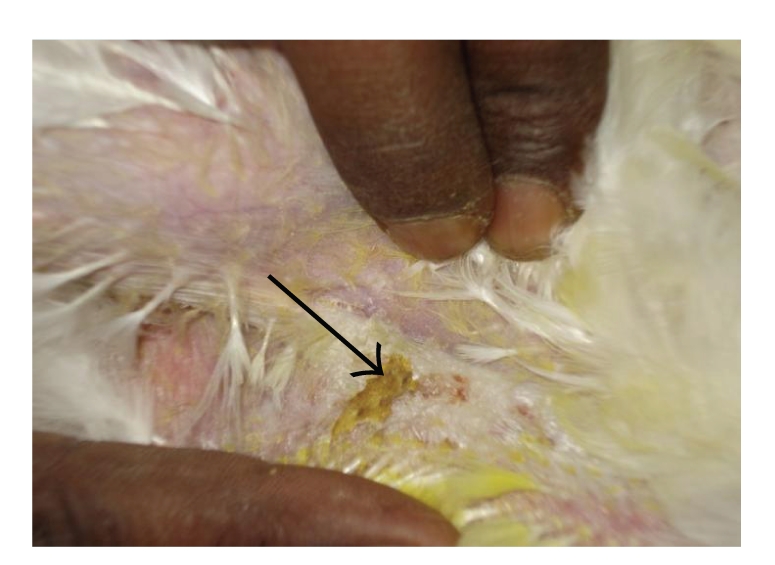
DTH reaction of skin (T-2 toxin plus SBT 400 ppm): mild increase in skin thickness and scab formation compare to toxin fed bird.

**Figure 7:**
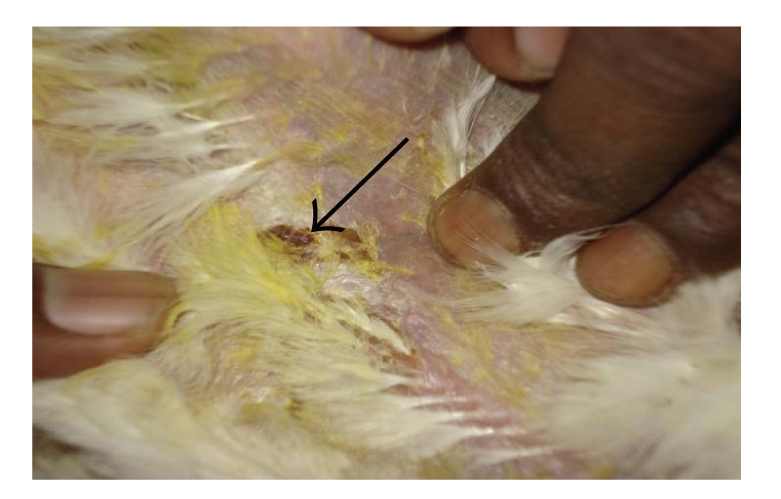
DTH reaction of skin (T-2 toxin plus SBT 800 ppm): moderate increase in skin thickness, ulceration, and scab formation compare to toxin-fed bird.

**Figure 8:**
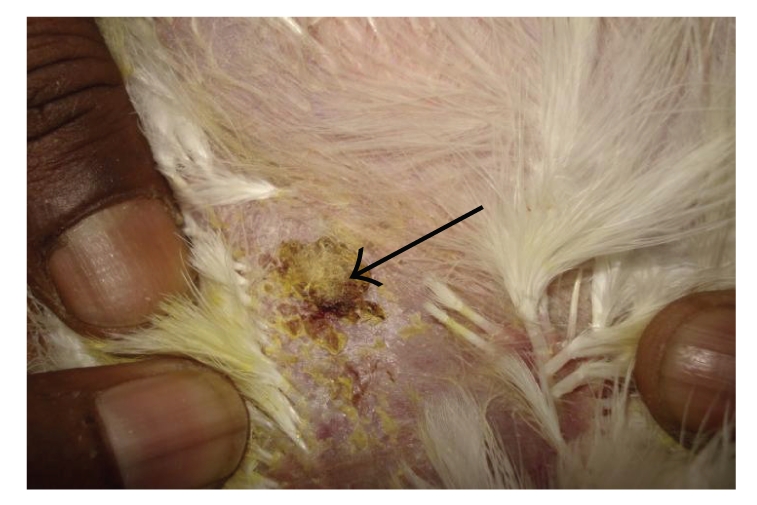
DTH reaction of skin (T-2 toxin plus SBT 400 ppm plus GM): severe increase in skin thickness, ulceration, induration, and scab formation compare to toxin-fed bird.

**Figure 9:**
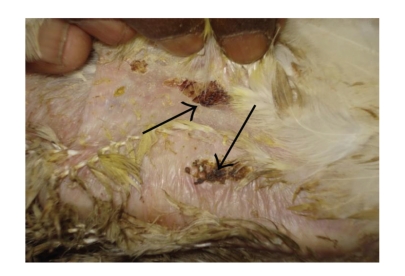
DTH reaction of skin (T-2 toxin plus SBT 800 ppm plus GM fed bird): severe increase in skin thickness, erythema, ulceration, induration, and large area of scab formation compare to toxin fed bird.

Grossly, the skin lesions were more severe and pronounced in SBT 800 ppm plus GM plus toxin-treated birds (Figure 9) as compared to healthy controls (Figure 3). The changes were relatively moderate in the birds treated with SBT 400 ppm plus GM plus toxin (Figure 8) and SBT 800 ppm plus toxin (Figure 7). These changes were mild in the birds treated with toxin plus GM (Figure 5) and toxin plus SBT 400 ppm (Figure 6). These changes were however, minimal in toxin only treated birds (Figure 4). The lesions were characterized by hot and painful focal swelling, erythema, induration, ulceration, and scab formation on the abdomen at 24 hrs after challenge with DNFB. Later on, these lesions exhibited the tendency to decrease at 36, 48 and 72 hrs in all the treatment groups.

## 5. Discussion

Mycotoxins, particularly trichothecene toxins, have been declared as immunosuppressive compounds in animals [1]. However, in previous studies, T-2 toxin had no effect on antibody production when administered orally or parenterally to chickens [13]. However, in the present study, feeding T-2 toxin to chickens resulted in a significant reduction in HI titres in both vaccinated (Newcastle disease virus) and unvaccinated birds. This might be due to the inherent ability of T-2 toxin to inhibit the protein synthesis through inhibition of peptidyl transferase activity [2]. The modified glucomannan significantly improved antibody titres against Newcastle disease virus, indicating that glucomannan probably reduces the potency of T-2 toxin or effectively binds aflatoxin [14]. The above results strongly support the significant increase observed in haemagglutination titres of birds receiving glucomannan, as compared to toxin fed birds. Similarly, a significant increase in total immunoglobulins in serum was also observed in glucomannan fed birds compared to toxin group. This result implies that glucomannan probably modulates the immune response against T-2 toxin-induced immunotoxicity [15]. 

The dietary levels of seabuckthorn at 400 and 800 ppm significantly increased the humoral immune response in vaccinated (Newcastle disease vaccine) and unvaccinated birds compared to toxin fed group. In earlier reports increased humoral immune response due to high content of ascorbic acid in seabuckthorn fruits has been reported [16]. Nevertheless, in one of the earlier studies, it has been observed that aflatoxin did not change the immunoglobulin levels [17]. On the contrary, in the present study a significant decrease in total serum immunoglobulin level was observed. This could be attributed to the potent immunosuppressive effect of T-2 toxin.

The increase in total immunoglobulin in serum might be attributed to high contents of vitamin E in seabuckthorn berries. In earlier studies, it has been reported that the vitamin E influences the development of the immune system in chickens [18]. In agreement to these reports, a significant increase in both HI titres was observed in vaccinated (NCD vaccine) birds and total immunoglobulins level in vaccinated (Newcastle disease vaccine) and unvaccinated birds was observed following seabuckthorn + glucomannan + toxin treatment on day 28. 

The T-2 toxin-fed birds showed significant decrease in nitroblue tetrazolium positive cells, which is indicative of decreased phagocytic activity in macrophages. The reduction of nonspecific immunity in present study is consistent with the depression of both humoral and cellular immunity. Compared to toxin-fed birds, the glucomannan combined with toxin significantly has increased the macrophage functions. The glucomannan has been reported to potentiate the nonspecific immune response against T-2 toxin [15]. A significant increase in macrophage function test was found in seabuckthorn-fed birds at both dietary levels compared to birds receiving toxin only. Similarly, an increase in macrophage function and the serum lysosomal activity has been reported in the mice fed with seabuckthorn extracts [19]. 

A significant increase function of peritoneal macrophage observed in birds fed with seabuckthorn and glucomannan combined with toxin. The contact between particular antigen and phagocytic cells is important for initiation of phagocytosis. The contact can be brought about by transport of particles via the blood or lymph to the sites of phagocytic cells. The combination of seabuckthorn and glucomannan combinedly might have increased the number of cells present in the peritoneum as well as increased their functional capacity.

Cell-mediated immune response in chicken was assessed by DTH reaction in the present experiment. A significant reduction in skin thickness and mild gross lesions was observed in T-2 toxin-fed birds. These results might be due to the reduction of lymphocyte production in lymphoid organs like spleen, thymus and bursa and bone marrow which cause immunosuppressive effect of T-2 toxin in chickens. Similarly, reduction in delayed type hypersensitivity reaction of chicks by aflatoxin [20] reported. A significant increase in skin thickness and moderate gross lesion were observed in birds fed with glucomannan and the toxin. This result indicates that the glucomannan has an ability to modulate the immune response [15]. A significant increase in skin thickness, erythema, induration, ulceration and scab formation observed in seabuckthorn with toxin-treated groups might be due to increased T- and B-cell response [21]. Similarly, Geetha et al. [22] reported that the seabuckthorn fruits and leaf extract stimulated the lymphocyte proliferation, IL-2 and *γ*-IFN production which were indicative of enhanced cellular immunity. A significant increase in skin thickness, severe erythema, induration, and scab formation was observed in DTH reaction area of birds fed toxin with seabuckthorn and glucomannan as compared to healthy controls at 24, 36, and 48 hrs. The results, thus, indicate an additive effect of both seabuckthorn and glucomannan.

## 6. Conclusions

T-2 toxin-fed at a dietary concentration of 1 ppm significantly reduced both humoral and cellular immune response of chickens, and dietary inclusion of glucomannan at 1 g/kg feed significantly protected birds from the immunotoxic effects of T-2 toxin. Seabuckthorn (400 and 800 ppm) alone significantly improved the immune response against T-2 toxin-induced adverse effects. Seabuckthorn and glucomannan in combination, however, provided an additive protection against T-2 toxin induced immunotoxicity. These findings indicate that the seabuckthorn and glucomannan providing additive protective effect in T-2 toxin-fed birds.

## Figures and Tables

**Table 1:** Effect of dietary supplementation of seabuckthorn berries (SBT) and glucomannan (GM, mycotoxin binder) on haemagglutination inhibition titres (log_2_ values) in birds fed with T-2 toxin.

Days posttreatment		Control	Toxin	Toxin + GM	Toxin + SBT400	Toxin + SBT800	Toxin + SBT400 + GM	Toxin + SBT800 + GM
28	Unvaccinated	2.333^ac^ ± 0.211	1.167^b^ ± 0.167	2.000^bc^ ± 0.258	2.500^ac^ ± 0.224	2.833^ac^ ± 0.167	3.000^a^ ± 0.259	4.000^d^ ± 0.259
	Vaccinated	5.833^ac^ ± 0.307	3.667^b^ ± 0.211	5.333^c^ ± 0.211	5.833^ac^ ± 0.167	6.500^ad^ ± 0.224	6.833^d^ ± 0.167	7.833^e^ ± 0.167

Mean values ± SE (*n* = 6). Means bearing same superscripts in between the columns do not differ significantly at 5% level.

**Table 2:** Effect of dietary supplementation of seabuckthorn berries (SBT) and glucomannan (GM, mycotoxin binder) on total immunoglobulin (g/dl) in birds fed with T-2 toxin.

Days Posttreatment		Control	Toxin	Toxin + GM	Toxin + SBT400	Toxin + SBT800	Toxin + SBT400 + GM	Toxin + SBT800 + GM
28	Unvaccinated	1.189^ac^ ± 0.121	0.403^b^ ± 0.028	0.889^c^ ± 0.154	1.223^ac^ ± 0.182	1.359^ac^ ± 0.068	1.443^a^ ± 0.126	1.918^d^ ± 0.080
	Vaccinated	1.422^ac^ ± 0.092	0.704^b^ ± 0.088	1.154^c^ ± 0.036	1.476^ac^ ± 0.038	1.598^a^ ± 0.046	1.633^a^ ± 0.104	2.093^d^ ± 0.097

Mean values ± SE (*n* = 6), Means bearing same superscripts in between the columns do not differ significantly at 5% level.

**Table 3:** Effect of dietary supplementation of seabuckthorn berries (SBT) and glucomannan (GM, mycotoxin binder) on skin thickness (mm) in delayed-type hypersensitivity reaction in birds fed with T-2 toxin.

Hour	Control	Toxin	Toxin + GM	Toxin + SBT400	Toxin + SBT800	Toxin + SBT400 + GM	Toxin + SBT800 + GM
0	1.771^a^ ± 0.068	1.714^a^ ± 0.046	1.843^a^ ± 0.020	1.800^a^ ± 0.044	1.686^a^ ± 0.059	1.757^a^ ± 0.043	1.671^a^ ± 0.029
12	3.171^ac^ ± 0.185	1.814^b^ ± 0.177	2.586^c^ ± 0.116	2.871^ac^ ± 0.129	2.986^ac^ ± 0.137	3.100^ac^ ± 0.244	3.557^a^ ± 0.138
24	3.371^a^ ± 0.200	1.929^b^ ± 0.149	2.643^c^ ± 0.102	3.471^a^ ± 0.068	3.614^a^ ± 0.110	3.743^a^ ± 0.057	4.329^d^ ± 0.171
36	2.743^ac^ ± 0.172	1.800^b^ ± 0.118	2.457^c^ ± 0.131	2.814^ac^ ± 0.059	2.886^acd^ ± 0.132	2.943^acd^ ± 0.143	3.414^d^ ± 0.110
48	2.186^ac^ ± 0.116	1.671^b^ ± 0.113	1.900^cb^ ± 0.079	2.429^ad^ ± 0.081	2.486^ad^ ± 0.163	2.571^ad^ ± 0.092	2.729^d^ ± 0.099
72	1.771^ab^ ± 0.124	1.543^b^ ± 0.166	1.800^ab^ ± 0.087	2.029^ab^ ± 0.115	2.129^ab^ ± 0.154	2.229^a^ ± 0.201	2.329^a^ ± 0.141

Mean values ± SE (*n* = 7), Means bearing same superscripts in between the columns do not differ significantly at 5% level.
